# Surgical Endodontics *vs* Regenerative Periodontal Surgery for Management of a Large Periradicular Lesion

**DOI:** 10.22037/iej.v13i2.20648

**Published:** 2018

**Authors:** Saeed Asgary, Leyla Roghanizadeh, Ali Haeri

**Affiliations:** a *Iranian Center For Endodontic Research, Research Institute of Dental Sciences, Dental School, Shahid Beheshti University of Medical Sciences, Tehran, Iran*

**Keywords:** Apical Periodontitis, Apical Seal, Calcium-Enriched Mixture, CEM Cement, Diagnostic Errors; Endodontic-Periodontal Lesions, Endodontic Surgery

## Abstract

Treatment success of periodontal-endodontic lesions is dependent on the elimination of both disease causative factors, whether they exist separately or concurrently. This report presents successful endodontic management of a misdiagnosed large periradicular pathology, which had not resolved after a previous periodontal regenerative surgery. A patient complaining of discomfort in the left maxillary region was referred. He had undergone regenerative surgery for treatment of a large periradicular defect; however, there was no further amelioration of the clinical signs/symptoms. Radiographically, a large periradicular lesion filled with bone substitute materials was detected around tooth #25. The endodontic treatment of the tooth was imperfect; therefore, surgical endodontic retreatment was planned. During root-end surgery, the biopsy containing bone substitute materials was obtained. Root-end filling/sealing using calcium-enriched mixture cement was completed. The histopathological examination showed granulation tissues enclosing exogenous materials. In two-year radiographic evaluation, resolving lesion and complete bone healing was observed. The first fundamental step in the management of periradicular lesions is correct diagnosis of the lesion origin and set-by step of the treatment plan according to the main causative factor. Regenerative periodonttal surgery, without considering the defective apical seal, will only cause a painful procedure for the patient without any positive benefit. Following appropriate apical seal, the endodontic lesion healing can be anticipated.

## Introduction

In differential diagnoses of periradicular lesions, the lesions with primary endodontic origin have the major contribution. However, the clinicians should also consider lesions of non-endodontic origin, including anatomic variations, other odontogenic or developmental cysts or neoplasms, and different categories of combined periodontal-endodontic lesions. Each one needs different treatment plan and has different prognosis [1, 2].

The pulp and periodontium are anatomically and functionally interrelated from the embryonic period to all over the life, in health and disease [3]. Endodontic-periodontal lesions, their diagnosis, management and prognosis have been one of the challenging issues in dental practice [4].

In primary endodontic lesions, resorption of the adjacent periapical bone and destruction of the attachment apparatus would happen. The suppurative process may establish a sinus tract that can extend through the periodontal ligament space and apical foramen. In such lesions with secondary periodontal involvement, consequent to a non-healed endodontic lesion, and as the result of continuing drainage and massing of plaque and calculus in the pocket, the periradicular alveolar bone would destroy further and can proceed into more apical migration of the attachment and establishing periodontal disease [5].

**Figure 1:**
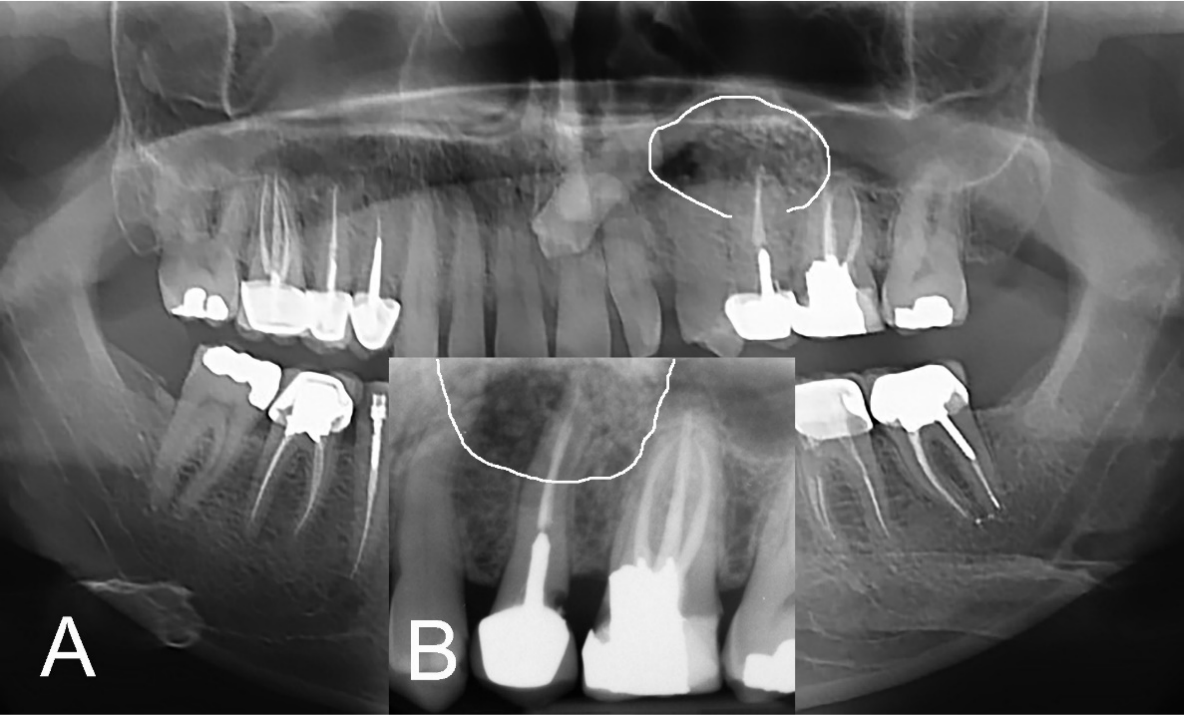
Primary radiographic evaluation, diffuse regenerative materials and unhealed periradicular lesion (white lines) around the endodontically treated root of the maxillary left second premolar; *A)* Panoramic radiograph, and *B)* Periapical radiograph

While a radiolucent lesion surrounds a previously root-treated tooth, the diagnosis and the treatment plan can be more complicated [6]. In the cases of failed conventional root canal treatment, nonsurgical retreatment can be the first choice. When nonsurgical retreatment is not practical or is predicted to have low success rate, the treatment plan would be surgical endodontics [7]. In this procedure, the root-end filling biomaterial would be inserted in the prepared root-end cavity that would close and seal the communication pathway through which the inflammatory mediators and pathogens could exchange [8].

This report presents successful management of a misdiagnosed large periradicular pathology. First, a periodontist intended to treat this lesion by a regenerative surgical procedure. As the operation outcome was not successful, the patient sought for treatment again.

## Case Report

A 30-year old man with frequent pain, discomfort and swelling in the left maxillary premolar area was referred to a private clinic. The patient stated that he had sought treatment for the problem by visiting a periodontist 6 months before. The specialist had performed a regenerative periodontal surgery in the region which resulted in no symptom relief. 

**Figure 2:**
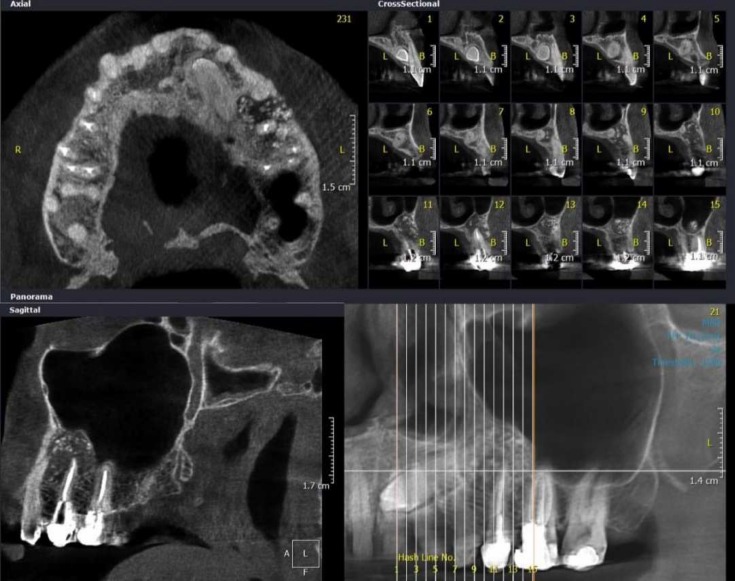
Preoperative cone-beam computed tomography evaluation: axial and cross sectional sections (above) and sagittal views (below) of the extending up periradicular lesion surrounding the root of tooth #25; the presence of bone replacement materials is noticeable

In clinical evaluation, the tooth #24 showed no caries and had normal response to pulpal sensibility tests. It had normal probing depth and was not sensitive to percussion. Tooth #25 had a metal-ceramic crown. It was obviously painful on percussion and the adjacent vestibule was so sensitive to palpation. The gingival mucosa showed slight swelling and redness. He had also complaint about chewing with the tooth. Probing examination showed normal attachment and depth (≤3mm) and no mobility was observed. In radiographic assessments (Figure 1A and B), a large periradicular lesion containing bone substitute materials around the root of the tooth #25 could be observed. The involved tooth had an inappropriate root canal treatment and a casting post and core. In cone-beam computed tomography evaluation, the periradicular radiolucency with the mean area size measured 1.4×1.5×1.7 mm, and the previously replaced bone substitutes could be observed (Figure 2).

Because of the surgical history, poor quality of the root canal therapy and existence of a casting post and core, the treatment plan decided to be a surgical endodontic retreatment. It was discussed with the patient and an informed consent was obtained. 

**Figure 3:**
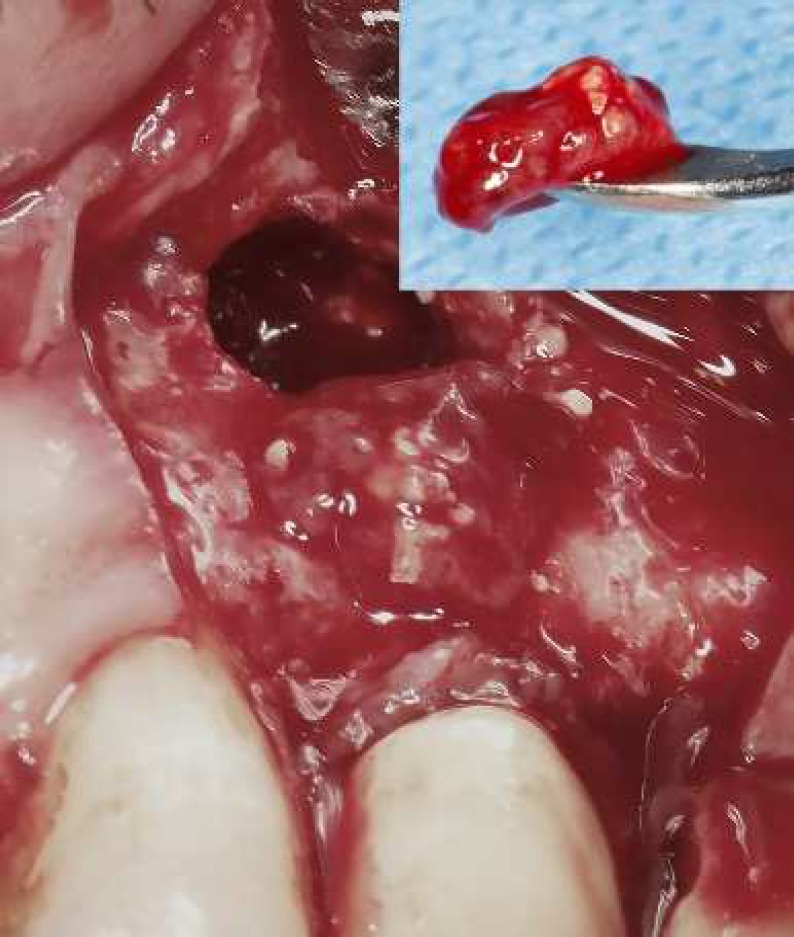
The curetted sample from periradicular lesion

An endodontist carried out the surgery. Following local anesthesia with 2% lidocaine with 1:80000 epinephrine (DarouPakhsh, Tehran, Iran), a full mucoperiosteal flap was raised. After flap reflection, the lesion was partially curetted to remove the inflamed tissues containing bone substitute (Figure 3) and gaining an appropriate access to the root tip. The curetted sample immersed in 10% formalin solution for submitting to an oral pathologist.

After root-end resection, the root-end cavity was prepared with an ultrasonic retrotip (Joya Electronics, Tehran, Iran).

**Figure 4:**
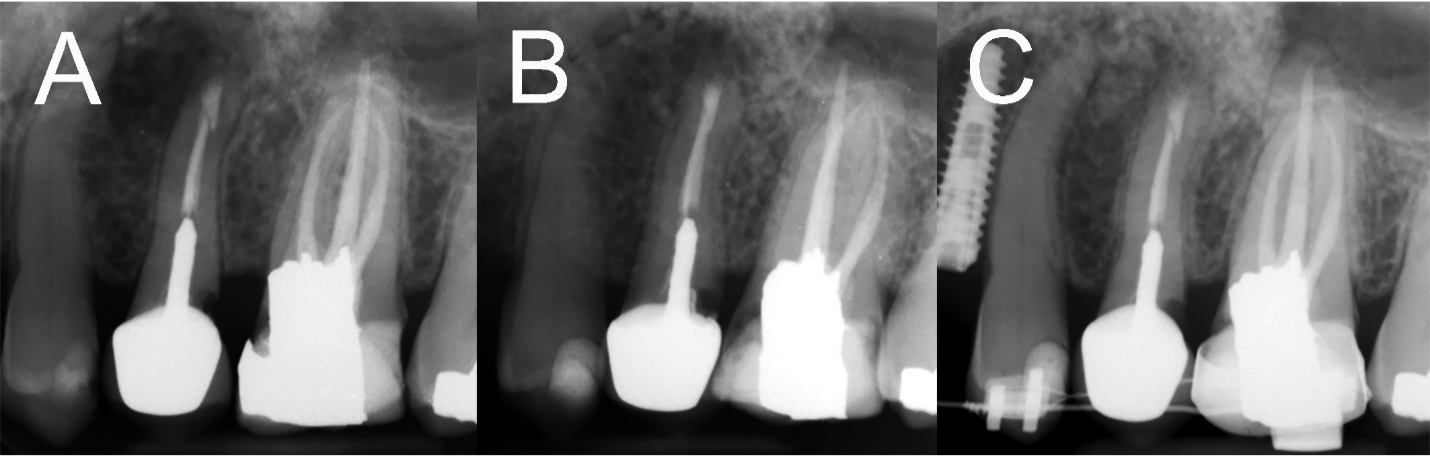
Postoperative radiographic examinations; *A*) Immediately after root-end filling; *B*) 6 months later, healing in progression; *C*) Two-year follow-up, radiographic assessment demonstrated recovery from the lesion and new bone formation

**Figure 5:**
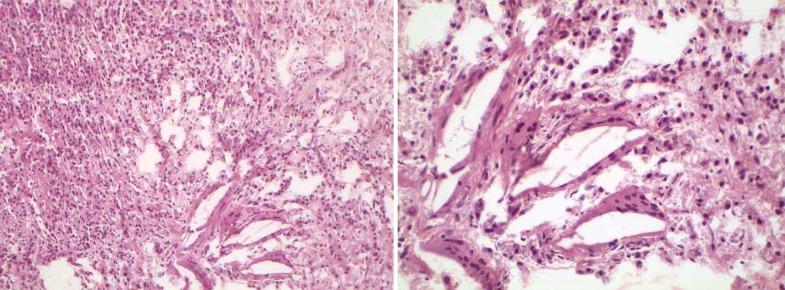
The histopathological evaluation revealed inflammatory infiltration and synthetic regenerative biomaterial

Calcium-enriched mixture (CEM) powder and liquid (BioniqueDent, Tehran, Iran) were mixed according to the manufacturer’s instructions. The biomaterial was inserted into the prepared cavity to achieve root-end filling/sealing. Subsequent to taking a confirmation radiography (Figure 4A), the flap was replaced and sutured. Histopathological examination verified granulation tissue with chronic inflammation enclosing the exogenous materials (Figure 5). 

The patient was recalled 7 days later. Clinical assessments showed absence of signs/symptoms, and the patient did not have any complaint about chewing with the tooth. On 6-month follow-up, the tooth was asymptomatic and functional. Healing of the lesion was uneventfully in progression (Figure 4B). On two-year follow-up visit, resolving the lesion, normal periodontal apparatus and new bone formation could be observed in the periapical radiography (Figure 4C).

## Discussion

This case report describes management of a large periradicular periodontitis, first misdiagnosed and mismanaged. Correct diagnosis is the crucial prerequisite for determining treatment strategies and long-term prognosis [9]. On some occasions in dental practice, differential diagnosis of well-defined radiolucencies surrounding teeth roots can be difficult [6]. To resolve a periradicular lesion, finding the origin of the lesion is the most important step. When encountering indefinite findings, non-endodontic lesions should be carefully distinguished from lesions with endodontic origin [10-12]. Therefore, to avoid misdiagnoses, the clinician should notice all of the information gathered from clinical and paraclinical examinations, also patient’s past medical and dental histories [1]. Sometimes histopathological evaluation is necessary for correct decision making [10]. Lesions of endodontic origin are raised as the result of the dental pulp necrosis [13]. Once inflammation and infection in the dental pulp begin, the immune defense mechanisms are stimulated to protect the host which mediate the mechanisms of humoral/cellular immunity [14]. However, in periradicular lesions of endodontic origin, as the microorganisms exist in a protected reservoir inaccessible to the immune system components, a challenge is imposed to the host defense. Conversely, invading the periodontium by pathogenic bacteria seems to be less challenging, and the host response may control the disease progression. Both diseases provoke inflammatory reactions which promote osteolytic alterations and mediate inhibition of bone formation [15].

Nevertheless, the primary endodontic lesions often cure following disinfection and sealing the root canal system; and one-year follow-up radiographic examinations usually demonstrates bone healing in the area [16]. Thus, it has been generally believed that in lesions with endodontic origin, if generalized periodontal disease has not been confirmed and the root has not any crack or fracture, a standard endodontic treatment would lead to healing of bone and other compartments of periodontium; however, there has been a discussion about the endodontic treatment effects on periodontal healing [9]. Despite an investigation supporting the idea of inhibitory effect of endodontic treatment on healing potential of the periodontium [17]; another study have shown that appropriate endodontic therapy have no significant influence on the healing of furcation defects [18].

The reported case was about a periradicular inflammatory odontogenic lesion with endodontic origin. There is similarity in clinical and radiographic aspects of primary endodontic disease with secondary periodontal involvement, primary periodontal disease with secondary endodontic involvement and true combined pathologies [19]. In spite of the above fact, often it is not complicated to distinguish primary endodontic lesions from primary periodontal disease [9]. Here could be a presumption that the preiodontist had not assessed the quality of previous endodontic treatment and performed the surgery without considering the apical seal. Therefore, the case was a diagnostic dilemma and a therapeutic challenge. 

What initiates periodontal disease is the microbial dental plaque and clinical investigations present the improvement in periodontitis resulting from improved plaque control; thus, accumulation of plaque and calculus and poor oral hygiene are the main environmental causative factors for periodontitis [20, 21]. The reported patient had good plaque control and oral hygiene with normal probing depth in all sites. Therefore, the lesion could not be diagnosed as a primary periodontal lesion. There are other reported lesions with endodontic origin in the literature which were initially had a periodontal graft surgery or planned to be extracted as suspected to be periodontal furcation involvement, severe periodontal bone loss or vertical root fracture [19, 22].

It can be assumed that maybe the previous insertion of bone substitute materials (synthetic filling materials) in the presented case might provide the matrix for the new bone regeneration in the region after elimination of the etiologic factors. Non autogenous bone replacement grafts can provide significant clinical improvements in osseous defects in comparison to surgical debridement alone [23]. In some patients, regeneration of the lost supporting structures could be seen after grafting intra-bony defects with bone replacement materials [24]. However, according to other studies, which evaluated the efficacy of guided tissue regeneration or placement of a covering membrane during endodontic periapical surgeries, these surgical procedures have no beneficial effect on bone formation or the rate of healing and the added charge would not be warranted in these cases [25-27]. In contrast, it was observed clinically, radiographically and histologically in another investigation that the simultaneous use of a non-bioabsorbable membrane and a synthetic bioactive resorbable graft resulted in complete regeneration of periapical bone defects. That bone regeneration could be attributed to first, the membrane permitted the re-population of the defect with regenerative cells derived from the endosteum and the periodontal ligament; and second, the filling material had the role of a reservoir and scaffold for the deposition of new bone [28].

An ideal root-end filling biomaterial should be able to create a three-dimensional seal and promote cementogenesis [29, 30]. Many studies on CEM cement revealed that the biomaterial is able to stimulate osteogenesis [30], dentinogenesis [31] and cementogenesis [8, 32]; in addition, the biomaterial create an effective seal against bacterial microleakage [33, 34]. When used as root-end filling, CEM cement is associated with regenerative periapical tissue response.

## Conclusion

To treat a large odontogenic lesion, the first important step is precise diagnosis of the lesion aetiology. Regenerative periodontal surgery, without considering the imperfect endodontic seal, might lead to treatment failure. Following appropriate apical seal using new root-end filling biomaterials, healing of the lesion can be predicted. 

## References

[1] Pontes FSC, Fonseca FP, de Jesus AS, Alves ACG, Araújo LM, do Nascimento LS (2014). Nonendodontic lesions misdiagnosed as apical periodontitis lesions: series of case reports and review of literature. J Endod.

[2] Rotstein I, Simon JH (2000). Diagnosis, prognosis and decision‐making in the treatment of combined periodontal‐endodontic lesions. Periodontol.

[3] Raja Sunitha V, Emmadi P, Namasivayam A, Thyegarajan R, Rajaraman V (2008). The periodontal–endodontic continuum: A review. J Conserv Dent.

[4] Rotstein I, Simon JH (2006). The endo‐perio lesion: a critical appraisal of the disease condition. Endod Topics.

[5] Cohen S, Hargreaves KM, Berman L (2011). Cohen's pathways of the pulp.

[6] Pace R, Cairo F, Giuliani V, Prato L, Pagavino G (2008). A diagnostic dilemma: endodontic lesion or odontogenic keratocyst? A case presentation. Int Endod J.

[7] Danin J, Strömberg T, Forsgren H, Linder LE, Ramsköld LO (1996). Clinical management of nonhealing periradicular pathosis: surgery versus endodontic retreatment. Oral Surg Oral Med Oral Pathol Oral Radiol Endod.

[8] Asgary S, Ehsani S (2013). Periradicular surgery of human permanent teeth with calcium-enriched mixture cement. Iran Endod J.

[9] Aksel H, Serper A (2014). A case series associated with different kinds of endo-perio lesions. J Clin Exp Dent.

[10] de Freitas Silva BS, Yamamoto-Silva FP, Sena-Filho M, Sant’Ana SSS, Mariano-Júnior WJ, de Almeida OP (2017). 20-year Follow-up of Recurrent Glandular Odontogenic Cyst Mimicking a Periapical Lesion. J Endod.

[11] de Carvalhosa AA, Zandonade RMC, de Araújo Estrela CR, Borges ÁH, Estrela C (2014). 8-year follow-up of central giant cell lesion mimicking apical periodontitis. J Endod.

[12] Bueno MR, De Carvalhosa AA, Castro PHDS, Pereira KC, Borges FT, Estrela C (2008). Mesenchymal chondrosarcoma mimicking apical periodontitis. J Endod.

[13] Nair P (2004). Pathogenesis of apical periodontitis and the causes of endodontic failures. Crit Rev Oral Biol Med.

[14] Martinho FC, Chiesa WM, Leite FR, Cirelli JA, Gomes BP (2012). Correlation between clinical/radiographic features and inflammatory cytokine networks produced by macrophages stimulated with endodontic content. J Endod.

[15] Graves DT, Oates T, Garlet GP (2011). Review of osteoimmunology and the host response in endodontic and periodontal lesions. J Oral Microbiol.

[16] Jivoinovici R, Suciu I, Gheorghiu I, Suciu I (2017). Clinical radiological aspects of primary endodontic lesions with secondary periodontal involvement. J Med Life.

[17] Sanders JJ, Sepe WW, Bowers GM, Koch RW, Williams JE, Lekas JS (1983). Clinical evaluation of freeze-dried bone allografts in periodontal osseous defects: Part III Composite freeze-dried bone allografts with and without autogenous bone grafts. J Periodontol.

[18] de Miranda JLC, Santana CMM, Santana RB (2013). Influence of Endodontic Treatment in the Post‐Surgical Healing of Human Class II Furcation Defects. J Periodontol.

[19] Asgary S, Fazlyab M (2014). Management of failed periodontal surgical intervention for a furcal lesion with a nonsurgical endodontic approach. Restor Dent Endod.

[20] Kinane DF, Peterson M, Stathopoulou PG (2000). Environmental and other modifying factors of the periodontal diseases. Periodontol.

[21] Stabholz A, Soskolne WA, Shapira L (2000). Genetic and environmental risk factors for chronic periodontitis and aggressive periodontitis. Periodontol.

[22] Lim J-H, Lee J-H, Shin S-J (2014). Diagnosis and treatment of teeth with primary endodontic lesions mimicking periodontal disease: three cases with long-term follow ups. Restor Dent Endod.

[23] Reynolds MA, Aichelmann-Reidy ME, Branch-Mays GL, Gunsolley JC (2003). The efficacy of bone replacement grafts in the treatment of periodontal osseous defects A systematic review. Ann Periodontol.

[24] Camelo M, Lynch SE, Nevins M Evaluation of Periodontal Regeneration Following Grafting Intrabony Defects with Bio-Oss Collagen: A Human Histologic Report. Int J Periodontics Restorative Dent.

[25] Santamaría J, García AM, de Vicente JC, Landa S, López-Arranz JS (1998). Bone regeneration after radicular cyst removal with and without guided bone regeneration: pathology. Int J Oral Maxillofac Surg.

[26] Garrett K, Kerr M, Hartwell G, O'sullivan S, Mayer P (2002). The effect of a bioresorbable matrix barrier in endodontic surgery on the rate of periapical healing: an in vivo study. J Endod.

[27] Taschieri S, Del Fabbro M, Testori T, Weinstein R (2007). Efficacy of xenogeneic bone grafting with guided tissue regeneration in the management of bone defects after surgical endodontics. J Oral Maxillofac Surg.

[28] Tobon S, Arismendi J, Marin M, Mesa A, Valencia J (2002). Comparison between a conventional technique and two bone regeneration techniques in periradicular surgery. Int Endod J.

[29] Utneja S, Nawal RR, Talwar S, Verma M (2015). Current perspectives of bio-ceramic technology in endodontics: calcium enriched mixture cement - review of its composition, properties and applications. Restor Dent Endod.

[30] Asgary S, Eghbal MJ, Ehsani S (2010). Periradicular regeneration after endodontic surgery with calcium-enriched mixture cement in dogs. J Endod.

[31] Asgary S, Nazarian H, Khojasteh A, Shokouhinejad N (2014). Gene expression and cytokine release during odontogenic differentiation of human dental pulp stem cells induced by 2 endodontic biomaterials. J Endod.

[32] Asgary S, Fazlyab M (2015). Surgical repair of invasive cervical root resorption with calcium-enriched mixture cement: a case report. Gen Dent.

[33] Hasheminia M, Loriaei Nejad S, Asgary S (2010). Sealing Ability of MTA and CEM Cement as Root-End Fillings of Human Teeth in Dry, Saliva or Blood-Contaminated Conditions. Iran Endod J.

[34] Nosrat A, Asgary S, Eghbal MJ, Ghoddusi J, Bayat-Movahed S (2011). Calcium-enriched mixture cement as artificial apical barrier: A case series. J Conserv Dent.

